# Supplementary material for: Social context suppresses food anticipatory activity and associated thermoregulation in mice
Source: PLoS One. 2026 Aug 28;21(8):e0356499. doi: 10.1371/journal.pone.0356499 (PMC13524294; doi:10.1371/journal.pone.0356499)
Supplement: Supplementary file 2 [file pone.0356499.s004.docx]

PONE-D-26-20478
Social Context Suppresses Food Anticipatory Activity and Associated Thermoregulation in Mice

Dear Dr. Foulkes

We thank the Academic Editor and Reviewers for their thoughtful and constructive evaluation of our manuscript. We are pleased that the reviewers found the study to be technically sound and recognized the value of using individualized telemetry to investigate food anticipatory activity (FAA) under socially relevant housing conditions. In response to the comments, we have revised the manuscript. Specifically, we have (1) moderated several mechanistic interpretations to better distinguish observations from hypotheses, (2) expanded the Discussion to address the complexity of social housing, including potential contributions of social hierarchy, stress, and thermoregulatory factors, (3) clarified methodological details regarding housing conditions, food intake measurements, statistical analyses, and temperature calculations, (4) expanded our discussion of the observed hypothermic episodes in singly housed males, and (5) improved figure presentation, reporting, and compliance with PLOS ONE formatting and animal welfare requirements. We believe these revisions have strengthened the manuscript and appreciate the opportunity to revise our work.

Responses to the Editor and Reviewers are provided below in **bold font**.

Sincerely,

Andrew Steele (on behalf of all co-authors)

Dear Dr. Steele,

Thank you for submitting your manuscript to PLOS ONE. After careful consideration, we feel that it has merit but does not fully meet PLOS ONE’s publication criteria as it currently stands. Therefore, we invite you to submit a revised version of the manuscript that addresses the points raised during the review process.

This manuscript has now been reviewed by 4 experts in the field as well as the ScreenIT system. They all gave a generally positive evaluation: The use of an individualised telemetry system was seen as a important innovation for exploring the origin of Food Anticipatory Activity within a social context and all recognised the interest in and value of these findings however, they have also raised a series of concerns that need to be addressed before this work can be considered suitable for publication.

A few points as examples:

The complexity of the "social environment" in this work seems to have been "glossed over" to some extent. Group housing may have multiple, complex effects on animals including affecting stress levels as well as impacting on hierarchies. These points need to be considered and discussed in much more detail.

**We agree and have expanded the Discussion to acknowledge that social housing simultaneously alters multiple environmental and physiological variables, including social interactions, cage density, thermoregulatory opportunities, stress responses, and social hierarchy. We now explicitly state that the present study was designed to evaluate the overall effect of social housing on FAA rather than identify which specific aspects of the social environment are responsible for the observed effects.**

The observed hypothermia bouts were seen as a potentially important finding but these were not discussed or examined in detail. Furthermore, the merits of considering links between FAA and body temperature changes need to be discussed in more detail.

**We agree that this was interesting, but it was not anything that we noticed until after our studies were completed so we did not get a chance to follow up on it. We revised the discussion slightly, while avoiding additional speculation, to mention the potential relationship between hypothermia, energetic demand, thermoregulation, and FAA while acknowledging that the present study was not designed to determine causality between these phenomena.**

Care should be taken when extrapolating from the results. In some has verged on pure speculation.

**We apologize for getting “carried away” in over-interpretations in several places. We deleted an entire paragraph from the Discussion. We made small revisions elsewhere to limit speculation. Mechanistic explanations related to energetic demand and thermoregulation are now framed as hypotheses consistent with the data rather than direct conclusions.**

The presentation of Figures and some data can certainly be optimised.

**We agree. We changed the layouts of Figures 1-3, presenting behavioral data in a column and side-by-side presenting temperature data in a column.**

An ethics statement is needed in the Methods section.

**We have added an ethics statement to the Methods section including our protocol number and name of our approval board (IACUC).**

When submitting your revision, we need you to address these additional requirements.

1. Please ensure that your manuscript meets PLOS ONE's style requirements, including those for file naming. The PLOS ONE style templates can be found at

<https://journals.plos.org/plosone/s/file?id=wjVg/PLOSOne_formatting_sample_main_body.pdf> and

[https://journals.plos.org/plosone/s/file?id=ba62/PLOSOne_formatting_sample_title_authors_affiliations.pdf](https://nam11.safelinks.protection.outlook.com/?url=https%3A%2F%2Ftrack.editorialmanager.com%2FCL0%2Fhttps%3A%252F%252Fjournals.plos.org%252Fplosone%252Fs%252Ffile%253Fid%3Dba62%252FPLOSOne_formatting_sample_title_authors_affiliations.pdf%2F1%2F010f019ec8d8484a-b7f7cdad-9899-43e0-84d0-765745ec8047-000000%2FJq1XTn9ujchYvjHUD5Yy020nUFCFRG5FbH8kVLFoTdg%3D258&data=05%7C02%7Cadsteele%40cpp.edu%7C1f5078eedd134875e79c08deca7b78a4%7C164ba61e39ec4f5d89ffaa1f00a521b4%7C0%7C0%7C639170829395755199%7CUnknown%7CTWFpbGZsb3d8eyJFbXB0eU1hcGkiOnRydWUsIlYiOiIwLjAuMDAwMCIsIlAiOiJXaW4zMiIsIkFOIjoiTWFpbCIsIldUIjoyfQ%3D%3D%7C0%7C%7C%7C&sdata=JLHbRnm7CajwcYup982zLoHs0SgftBVUs1lxCuyLXpc%3D&reserved=0)

**We have adjusted level 1 and 2 headings, the referencing format as brackets, and believe that is the only style requirement that needed to be altered.**

2. To comply with PLOS One submissions requirements, in your Methods section, please provide additional information regarding the experiments involving animals and ensure you have included details on (1) methods of sacrifice, (2) methods of anesthesia and/or analgesia, and (3) efforts to alleviate suffering.

**We expanded the Methods section to include details regarding anesthesia, perioperative monitoring, efforts to minimize animal discomfort, and euthanasia procedures where applicable.**

3.  We note that the grant information you provided in the ‘Funding Information’ and ‘Financial Disclosure’ sections do not match.

When you resubmit, please ensure that you provide the correct grant numbers for the awards you received for your study in the ‘Funding Information’ section

**Funding information will be corrected upon resubmission. We are listing two funding sources total.**

4. Thank you for stating the following financial disclosure:

“Funding was provided by the National Institute of General Medical Sciences of the National Institutes of the National Institutes of Health under award number R16GM145576 and by the National Institute of Diabetes and Digestive and Kidney Diseases of the National Institutes of Health DK132736 to ADS”. **The funders had no role in study design, data collection and analysis, decision to publish, or preparation of the manuscript.**

Please state what role the funders took in the study.  If the funders had no role, please state: "The funders had no role in study design, data collection and analysis, decision to publish, or preparation of the manuscript."

Please include this amended Role of Funder statement in your cover letter; we will change the online submission form on your behalf.

**Completed above**

5. Please include your full ethics statement in the ‘Methods’ section of your manuscript file. In your statement, please include the full name of the IRB or ethics committee who approved or waived your study, as well as whether or not you obtained informed written or verbal consent. If consent was waived for your study, please include this information in your statement as well.

**The experiments described herein were approved by the California State Polytechnic, Pomona Institutional Animal Care and Use Committee (IACUC) under protocol 23.009.**

6. Please include captions for your Supporting Information files at the end of your manuscript, and update any in-text citations to match accordingly. Please see our Supporting Information guidelines for more information: [http://journals.plos.org/plosone/s/supporting-information](https://nam11.safelinks.protection.outlook.com/?url=http%3A%2F%2Ftrack.editorialmanager.com%2FCL0%2Fhttp%3A%252F%252Fjournals.plos.org%252Fplosone%252Fs%252Fsupporting-information%2F1%2F010f019ec8d8484a-b7f7cdad-9899-43e0-84d0-765745ec8047-000000%2FDhhppZdfy_N2n-Vnr8q2-5hLw4WLmjEERh1kGobL1FY%3D258&data=05%7C02%7Cadsteele%40cpp.edu%7C1f5078eedd134875e79c08deca7b78a4%7C164ba61e39ec4f5d89ffaa1f00a521b4%7C0%7C0%7C639170829395768673%7CUnknown%7CTWFpbGZsb3d8eyJFbXB0eU1hcGkiOnRydWUsIlYiOiIwLjAuMDAwMCIsIlAiOiJXaW4zMiIsIkFOIjoiTWFpbCIsIldUIjoyfQ%3D%3D%7C0%7C%7C%7C&sdata=3K%2F71mbg143kBEXmAZ0zuUoJ0MgNV7uYOyABnMIK3%2Fw%3D&reserved=0).

**Supporting Information captions have been added and all in-text citations have been updated accordingly.**

Reviewers' comments:

Reviewer's Responses to Questions

**Comments to the Author**
1. Is the manuscript technically sound, and do the data support the conclusions?
The manuscript must describe a technically sound piece of scientific research with data that supports the conclusions. Experiments must have been conducted rigorously, with appropriate controls, replication, and sample sizes. The conclusions must be drawn appropriately based on the data presented.

Reviewer #1: Yes

Reviewer #2: Yes

Reviewer #3: Yes

Reviewer #4: Yes

Reviewer #5: Partly

2. Has the statistical analysis been performed appropriately and rigorously?

Reviewer #1: Yes

Reviewer #2: Yes

Reviewer #3: Yes

Reviewer #4: Yes

Reviewer #5: No

3. Have the authors made all data underlying the findings in their manuscript fully available?
The [PLOS Data policy](https://nam11.safelinks.protection.outlook.com/?url=http%3A%2F%2Ftrack.editorialmanager.com%2FCL0%2Fhttp%3A%252F%252Fwww.plosone.org%252Fstatic%252Fpolicies.action%2523sharing%2F1%2F010f019ec8d8484a-b7f7cdad-9899-43e0-84d0-765745ec8047-000000%2FB6s1_WludOc2_a9r9HQZz5PcA9GZSd4HGf1lvveTVWY%3D258&data=05%7C02%7Cadsteele%40cpp.edu%7C1f5078eedd134875e79c08deca7b78a4%7C164ba61e39ec4f5d89ffaa1f00a521b4%7C0%7C0%7C639170829395780672%7CUnknown%7CTWFpbGZsb3d8eyJFbXB0eU1hcGkiOnRydWUsIlYiOiIwLjAuMDAwMCIsIlAiOiJXaW4zMiIsIkFOIjoiTWFpbCIsIldUIjoyfQ%3D%3D%7C0%7C%7C%7C&sdata=RSodJ7SFb8sykDkzcivMaV6hZisqtEU9T%2B94Tiz4P38%3D&reserved=0) requires authors to make all data underlying the findings described in their manuscript fully available without restriction, with rare exception (please refer to the Data Availability Statement in the manuscript PDF file). The data should be provided as part of the manuscript or its supporting information, or deposited to a public repository. For example, in addition to summary statistics, the data points behind means, medians and variance measures should be available. If there are restrictions on publicly sharing data—e.g. participant privacy or use of data from a third party—those must be specified.

Reviewer #1: Yes

Reviewer #2: Yes

Reviewer #3: Yes

Reviewer #4: Yes

Reviewer #5: No

4. Is the manuscript presented in an intelligible fashion and written in standard English?
PLOS ONE does not copyedit accepted manuscripts, so the language in submitted articles must be clear, correct, and unambiguous. Any typographical or grammatical errors should be corrected at revision, so please note any specific errors here.

Reviewer #1: Yes

Reviewer #2: Yes

Reviewer #3: Yes

Reviewer #4: Yes

Reviewer #5: Yes

5. Review Comments to the Author
Please use the space provided to explain your answers to the questions above. You may also include additional comments for the author, including concerns about dual publication, research ethics, or publication ethics. (Please upload your review as an attachment if it exceeds 20,000 characters)

Reviewer #1: This manuscript presents an interesting and technically sound descriptive study demonstrating that group housing suppresses food anticipatory activity (FAA) and associated preprandial temperature rhythms in mice. The use of implanted telemetry devices enabling individual-level measurements in socially housed mice represents a notable methodological strength. However, several conceptual and presentation issues should be addressed before publication.

Major Comments

1. Observation versus mechanism. The data convincingly demonstrate that group housing attenuates FAA and associated thermal rhythms. However, several mechanistic interpretations currently extend beyond what is directly supported by the presented data. In particular, the discussion of thermoregulatory demand, energetic state, and integrated physiological mechanisms should be framed more cautiously as hypotheses or possible interpretations rather than demonstrated mechanisms. The present study is strongest as a descriptive physiological report, and the conclusions should remain closely aligned with the experimental evidence.

**We appreciate this comment and agree that some mechanistic interpretations were stated too strongly in the original version. We have revised the Discussion to better distinguish between observations and hypotheses. The manuscript now emphasizes that our primary finding is the suppression of FAA and associated temperature fluctuations under group housing conditions. Proposed explanations involving thermoregulatory demand and energetic state are now framed as interpretations consistent with the observed data rather than demonstrated mechanisms.**

2. Interpretation of “social context.” Group housing changes multiple environmental and physiological variables simultaneously, including social interaction, cage density, thermal microenvironment, stress levels, and potential dominance hierarchy effects. Therefore, the conclusions should be phrased carefully as effects of group housing/social housing conditions, rather than attributing the findings specifically to social interaction or thermoregulatory mechanisms alone.

**We agree that social housing alters multiple variables simultaneously. Throughout the revised manuscript we have replaced language implying a specific role for social interaction alone with language referring more broadly to social housing conditions. We have also expanded the Discussion to acknowledge potential contributions of social hierarchy, cage density, stress, and thermal microenvironment.**

3. Body temperature as an FAA-related parameter. The body temperature data are valuable and support the behavioral findings; however, body temperature should not be treated as equivalent to FAA itself. The manuscript would benefit from clearer distinction between locomotor FAA and associated physiological outputs such as preprandial thermal rhythms. In addition, because the study measures subcutaneous temperature rather than core body temperature, the limitations and potential influence of thermal microenvironment should be more clearly acknowledged.

**We agree and have revised the text to more clearly distinguish locomotor FAA from associated physiological outputs such as preprandial temperature rhythms. We also expanded discussion of the limitations of using subcutaneous temperature measurements and acknowledge that local thermal conditions may influence these measurements differently than core body temperature.**

4. Figure organization and temporal alignment. The current arrangement of Figure panels (A–N) makes comparison between locomotor activity and temperature somewhat difficult. A vertically aligned two-column layout (e.g., activity in one column and temperature in the other) may improve readability and better emphasize the parallel suppression of both outputs. In addition, the x-axis of panels C–J is currently centered around the nocturnal active phase. Since the principal biological phenomenon under investigation is FAA during ZT4–6, it would be more effective to center the representative traces around the preprandial anticipatory window.

**We thank the reviewer for this helpful suggestion. We have revised the figure to use a two-column layout, with locomotor activity and temperature panels aligned in parallel, which we agree improves readability and facilitates comparison between these outputs.**

**We also appreciate the reviewer’s suggestion regarding temporal alignment of the representative traces. After careful consideration, we chose to retain the current centering of the activity plots. In our prior work, including more than a dozen related publications, we have consistently displayed the pre-meal anticipatory window on the right side of the binned plots. Maintaining this convention allows for continuity with our previous studies and facilitates comparison across datasets. We agree that centering the plots around the FAA window is a reasonable alternative, but in this case we felt that preserving the established format was preferable.**

Minor Comments
1. “ZT Time” is redundant. Because ZT already stands for “Zeitgeber Time,” the figure labels should be revised to either “ZT” or “Zeitgeber Time.”

**Agreed, that has been fixed throughout the Figures.**

2. Statistical reporting. The manuscript is generally well analyzed statistically. However, consistent formatting of statistical notation and spacing throughout the manuscript would improve readability.

**We have adjusted spacing and ensured consistency with descriptions of statistical tests and their results.**

3. Language and presentation. The manuscript is generally well written in standard scientific English. Only minor editorial revisions are required.

**We went through the manuscript with a fine-toothed comb and made several grammatical edits.**

Reviewer #2: This is an automated report for PONE-D-26-20478. This report was solicited by the PLOS One editorial team and provided by ScreenIT.

**No concerns were raised.**

Reviewer #3: This manuscript addresses a genuine and underappreciated gap in circadian biology — the near-total absence of FAA studies conducted under socially relevant housing conditions. Using subcutaneously implanted wireless telemetry ("nanotags"), the authors tracked individual locomotor activity and body temperature in both singly and group-housed C57BL/6J mice during time-restricted feeding. The core finding — that social housing suppresses FAA and blunts preprandial temperature rises — is biologically meaningful and methodologically enabled by a clever use of individualized telemetry in a group setting. The paper is generally well-written and the logic is sound. However, several methodological and analytical issues warrant attention before acceptance.

Comment 1: Group composition is partially confounded
Each group-housed cage contained two implanted and two non-implanted mice. The non-implanted animals serve no described experimental purpose yet share the social and thermal environment. Their presence may influence the behavior of implanted animals (e.g., huddling partners, dominance dynamics), and the reader has no information about whether these "filler" mice received any monitoring. This design choice should be explicitly justified, and the authors should address whether the non-implanted mice were screened for health or weight changes throughout the study.

**We thank the reviewer for raising this important point. We have clarified the rationale for this design in the Methods section. In the group-housed condition, each cage contained two implanted mice and two non-implanted age-matched cage mates. This configuration was used to maintain a standard group-housed social environment while allowing behavioral and physiological monitoring of individually identified implanted animals.**

**We agree that the presence of non-implanted cage mates could influence the behavior of implanted animals through normal social interactions, including huddling or dominance-related behaviors. However, all group-housed cages were configured identically, with the same ratio of implanted to non-implanted animals. Thus, any influence of non-implanted cage mates was a consistent component of the group-housing condition rather than an uncontrolled difference among group-housed cages.**

**We did not record any data regarding the non-implanted mice. We now state this explicitly in the Methods and acknowledge this as a limitation of the study.**

Comment 2: Social hierarchy and individual variation are unaddressed
Group housing in male mice, particularly C57BL/6J, routinely produces dominance hierarchies that influence stress hormones, body weight, and activity levels. Given that 6/12 single-housed males showed hypothermic bouts — a striking degree of individual variation — it is unknown whether dominant vs. subordinate animals differ in FAA expression within group-housed cages. No within-cage individual comparisons are reported. This warrants at minimum an expanded discussion of how social hierarchy could confound or modulate the reported suppression of FAA in grouped animals.

**We agree that social hierarchy is a potentially important variable in group-housed mice and may contribute to variation in FAA expression. However, dominance status was not assessed in the present study and therefore we cannot determine whether dominant and subordinate animals differed in anticipatory activity or temperature rhythms. We have expanded the Discussion to acknowledge this limitation and note that future studies directly measuring social rank will be necessary to determine the contribution of hierarchy to FAA. Importantly, the objective of the present study was to determine whether social housing as a condition alters FAA rather than identify specific social mechanisms responsible for the observed effects.**

Comment 3: Delta temperature metric requires clearer justification
The authors define delta temperature as the maximum value within the ZT6 hour minus the minimum within the ZT24 hour, derived from 5-minute bins. This operationalization is sensitive to outliers within those one-hour windows, as a single anomalous bin could disproportionately influence the daily delta value. A brief justification for this approach over alternatives — such as hourly means or a broader averaging window — would improve methodological transparency and reproducibility.

**We agree in principle, but in practice, having looked at many temperature bins, we can say that there were not dips and spikes in any of the 5-minute bins (temperature doesn’t change that dramatically across 5 minutes). We also analyzed the data, taking the average temperature at ZT 6 and 24 and the graphs were very similar. We acknowledge that alternative approaches are possible and have clarified this rationale in the Methods of the revised manuscript.**

Comment 4: Figure 2C shows a significant difference on Day 0 before feeding restriction begins
The Results text describes Day 0 activity and temperature profiles as "broadly similar" between single and group-housed males, yet Figure 2D displays asterisks on the temperature trace, indicating statistically significant differences at baseline before any feeding manipulation was introduced. This discrepancy between the text and the figure is not adequately addressed in the Results section. Pre-existing baseline differences between housing groups could complicate the interpretation of subsequent changes as being driven by the time-restricted feeding protocol rather than by housing condition alone, and should be explicitly discussed.

**We thank the reviewer for identifying this discrepancy. We revised this description in the results to read: “Prior to the start of the scheduled feeding (Day 0), activity levels were strongly nocturnal and temperature profiles were consistently lower in single housed mice but also showed a clear elevation at night…”**

Comment 5: Figure 1N legend contains an error
The legend for Figure 1N describes the delta temperature metric as "ZT24–7," while the Methods section defines it as the maximum temperature at ZT6 minus the minimum at ZT24, and Figure 2N correctly labels it "ZT24–6." The endpoint in the Figure 1N legend should be corrected to ZT6 for consistency with the Methods and other figures.

**Corrected.**

Comment 6: The hypothermia finding is striking but underexplored
The observation that 6 out of 12 single-housed males exhibited acute hypothermic bouts (subcutaneous temperature below 28°C) during the mid-light phase is notable and reported with appropriate statistics via Fisher's exact test. However, this finding receives only brief mention and is relegated to a supplemental figure. Key questions remain unaddressed: Do these animals show correspondingly stronger FAA? Does hypothermia precede or follow anticipatory activity bouts, suggesting an energetic cost of FAA in isolated animals? This finding has the potential to meaningfully support the paper's central argument that FAA reflects an energetically demanding physiological state, and it deserves integration into the main Discussion rather than remaining in supplemental material alone.

**We agree that this observation is potentially important, but we also did not want it to distract from the overall message of the manuscript, which is that social housing conditions are generally suppressive of food anticipatory activity and involve a less severe change in body temperature. The hypothermia is also transient: it was not present throughout the experiment. The mice with severe hypothermia had dramatically reduced activity overall as could be expected from a torpor state. We felt that their lack of FAA was likely trivial in nature (i.e. if an animal is in torpor, then it cannot show FAA). As we have already been warned by the editor and reviewers to tone down our speculations, I do not think it is prudent to discuss this in the manuscript as it is speculative.**

Reviewer #4: Manuscript ID: PONE-D-26-20478

This is a very interesting manuscript reporting food-anticipatory locomotor activity and subcutaneous body temperature in social-housed conditions in mice. The authors measured individual locomotor activity and body temperature changes in response to timed-food restrictions in single-housed and group-housed mice using wireless implants. The anticipatory locomotor activity was suppressed in group-housed mice.
I believe the study of the effect of social housing on anticipatory behavior is interesting and warrants publication in the journal. However, I have a few concerns.

**We thank the reviewer for raising this point and for noting the apparent increase in food intake during the restricted feeding period relative to Day 0. Day 0 corresponds to the first day of restricted feeding. On this initial day, mice had not yet adapted to the restricted feeding schedule and therefore consumed relatively little during the 3-hour food access window. On subsequent RF days, intake increased as the mice adapted to the predictable timing of food availability. However, because food was available for only 3 hours, intake remained limited to approximately 3 g per mouse during the feeding window, consistent with an overall calorie-restricted condition relative to ad libitum feeding.**

**Food intake values shown for Days 0, 7, 14, and 20 represent intake measured during the restricted feeding window on those specific days, rather than a 7-day average. For group-housed cages, food intake was measured at the cage level and normalized by the number of animals in the cage to generate per-animal estimates. We have clarified these details in the Methods section.**

**We agree that measuring intake individually in group-housed mice would have provided a more precise estimate of individual food consumption. However, separating mice during the feeding period would have disrupted the group-housed condition and potentially altered feeding behavior. We therefore chose to preserve the social housing condition and report cage-level intake normalized per animal.**

Minor comments:
1. Include the ethics statement in the material and methods section

**Added as requested.**

2. Adding the feeding window in the figures, either as a bar below the graph or as a distinct shaded area, would make it easier for the readers to interpret the data.

**Response: We thank the reviewer for this suggestion. We added a brown bar at ZT 7-9 to indicate the period of food availability.**

Reviewer #5: This is an interesting and potentially publishable study. It addresses an important gap by testing whether social housing alters food anticipatory activity and related thermoregulation in mice, using implanted nanotags to monitor individuals in group-housed cages. The main finding, that group housing suppresses FAA especially in males, is clear and relevant.

However, several issues require clarification before the conclusions can be fully supported.

1. Lines 368–375. The main concern is whether individual mice or cages should be treated as the experimental unit. Although nanotags provide individual-level measurements, mice within the same group-housed cage share food access, social environment, microclimate, hierarchy, odor cues, and handling. The authors state that group-housed cages contained four mice and that individual animals were used as the unit of analysis, but cage may be the appropriate unit for the housing manipulation. The authors should report the number of cages per group and either reanalyze key outcomes using cage as a random effect or cage-level means, or justify animal-level independence.

**We appreciate the reviewer's concern regarding pseudoreplication in group-housed studies. In the present work, locomotor activity and body temperature were measured continuously from individual mice using implanted nanotags, allowing each animal's physiological and behavioral responses to be quantified independently. We acknowledge that animals within a shared cage are not completely independent with respect to housing condition and have clarified this in the Methods section as a limitation. However, because the primary outcomes analyzed in this study were individual-level physiological and behavioral measurements rather than cage-level measurements, we believe treatment of the animal as the experimental unit is appropriate and consistent with previous telemetry-based behavioral studies.**

2. Lines 376–380. Food intake is used to argue that caloric intake does not drive the phenotype, but in group-housed cages intake appears to have been measured per cage rather than per individual. Authors should clarify how intake was measured and normalized, and whether implanted and non-implanted animals had comparable food access.

**We agree that clarification was needed. Additional details regarding food intake measurements and normalization procedures have been added to the Methods section. Please also see our response to Reviewer 4 above, as they raised a similar concern.**

3. Lines 143–150. Group-housed males weighed more than singly housed males despite similar reported food intake. This finding should be discussed more explicitly, as it may support the energetic-demand model but also raises questions about thermogenic costs, hypothermia, anticipatory activity, or unequal food access.

**We agree that the greater body weight observed in group-housed males despite similar food intake is an interesting finding. We have expanded the Discussion to consider the possibility that reduced thermoregulatory demands associated with social housing may contribute to improved energy conservation and body weight maintenance. We also acknowledge that additional factors associated with social housing may contribute to this phenotype.**

4. Lines 160–164. The hypothermic bouts in singly housed males are potentially important. The authors should define these events more rigorously, report their duration and frequency, and test whether animals showing hypothermia disproportionately drive the FAA phenotype. A sensitivity analysis excluding these animals would be useful.

**We have expanded our discussion of these hypothermic episodes and their potential physiological significance in the same Discussion paragraph where we respond to point 3 just above. However, believe definitive evaluation of this possibility will require future studies.**

5. Lines 198–228. The sex-specific interpretation should be softened. The abstract states that social housing suppresses FAA in both sexes, but the female effect appears weaker and transient, whereas the male effect is more robust and sustained. The abstract and discussion should reflect this distinction.

**We agree and have revised the Abstract to more clearly reflect this distinction. The sentence now reads: “Social housing significantly suppressed FAA in both male and female mice, with the effect being more pronounced and sustained in males.” We also reviewed the Discussion and believe the current wording already presents a cautious interpretation of the sex-specific effects. In particular, the Discussion notes that the suppression of FAA was more pronounced and sustained in males, whereas females showed a more modest and transient reduction during the early phase of restricted feeding. It also emphasizes that sexes were not directly compared in this study and that lower baseline FAA in females may limit the dynamic range available to detect housing-dependent suppression. Thus, the revised Abstract is now aligned with the more cautious interpretation already presented in the Discussion.**

6. Lines 401-410. The statistical section needs more detail. The authors should specify which variables were analyzed with ANOVA versus mixed-effects models, how repeated measures and missing data were handled, and whether assumptions were checked. A summary table of analyses would improve reproducibility.

**We have expanded the Statistical Analysis section to provide additional detail regarding the use of ANOVA and mixed-effects models, treatment of repeated measures, handling of missing values, and post hoc testing procedures. We have also clarified the rationale for selecting specific statistical approaches for individual analyses.**
