# Supplementary material for: Social context suppresses food anticipatory activity and associated thermoregulation in mice
Source: PLoS One. 2026 Aug 28;21(8):e0356499. doi: 10.1371/journal.pone.0356499 (PMC13524294; doi:10.1371/journal.pone.0356499)
Supplement: Supplementary file 10002 [file pone.0356499.s003.pdf]

ScreenIT Report

This is an automated report for 10.64898+2026.04.10.717725.pdf. This report was solicited by the PLOS One editorial team and provided by ScreenIT.

ScreenIT is an independent group of scientists developing automated tools that analyze academic papers. A set of automated tools screened your submitted manuscript and provided the report below. Each tool was created by your academic colleagues with the goal of helping authors.

The tools look for factors that are important for transparency, rigor and reproducibility, and we hope that the report might help you to improve reporting in your manuscript. Within the report you will find links to more information about the items that the tools check. These links include helpful papers, websites, or videos that explain why the item is important.

While our screening tools aim to improve and maintain quality standards they may, on occasion, miss nuances specific to your study type or flag something incorrectly. Each tool has limitations that are described on the ScreenIT website. The tools screen the main file for the paper; they are not able to screen supplements stored in separate files.

Please note that the Academic Editor had access to these comments while making a decision on your manuscript. The Academic Editor may ask that issues flagged in this report be addressed.

If you would like to provide feedback on the ScreenIT tool, please email the team at ScreenIt@bih-charite.de. If you have questions or concerns about the review process, please contact the PLOS One office at plosone@plos.org.

How to read the report:

- no action required

?- review for potential issues

!- a critical issue was found by a tool

n/a - may not be applicable to your paper

- more information about this item can be obtained using this link

Rigor

✓	Ethics	Euthanasia Agents: "Mice were anesthetized using isoflurane anesthesia and then a nanotag was implanted subcutaneously through a 2-cm incision on the dorsal midline."
?	Inclusion and Exclusion Criteria	Not detected. Please specify any pre-defined criteria used to determine whether participants, animals or samples were eligible for the study, or exclude participants, animals or samples from the study.
?	Flow charts and attrition	We did not find a study flow chart of excluded observations. We strongly recommend using flow charts because they provide an overview of the study design and more information about attrition. If you included a study flow chart in your supplemental files, we apologize for missing this. Our tool is not able to screen separate supplemental files.<br><br>Sentence about attrition: not detected. Please provide information about the drop-out of subjects, or loss of animals or samples. This could be done using a study flow chart, or described in the text.
✓	Sex as a biological variable	"Male and female mice were 1114 weeks of age at the start of restricted feeding."
✓	Subject Demographics	Age: "Male and female mice were 1114 weeks of age at the start of restricted feeding."
?	Randomization	Not detected. If you performed an experimental study, please specify whether participants, animals or samples were randomly assigned to treatments or groups, and specify the randomization procedure.
?	Blinding	Not detected. Please specify whether blinding was used at various phases of the experiment (e.g., blinding of patients, caregivers, outcome assessments, data analysis).
	Power Analysis	Not detected. Please state how you determined the sample size for the study. If you

		performed a power or sample size calculation, please provide details.
	Replication	not required.

Generated by Barzooka (flow charts), SciScore (everything else).
SciScore only screens the text of the methods section; we apologize if the tool has missed items reported elsewhere in the paper or supplements.

Resources

Persistent unique identifiers for research resources, RRIDs, can be used to track these key resources though the literature. They are requested or required in many journals, but even if your journal does not require them, we strongly encourage the use of these for the readers of papers who wish to easily track down the key materials like antibodies, software tools, or mice.

Experimental Models: Organisms/ Strains	"Food intake, body weight, locomotor activity, and body temperature were compared between male C57BL/6J mice housed in small (n=8) or large (n=12) cages during restricted feeding."	C57BL/6J suggested: RRID:IMSR_JAX:000664. Please make sure this RRID suggestion is accurate before including in your manuscript.
Software and Algorithms	"Statistical testing All statistical analyses were performed using GraphPad Prism (version 10.0; GraphPad Software)."	GraphPad Prism suggested: None. Please add an RRID to help others identify this resource.<br>GraphPad suggested: GraphPad Prism, RRID:SCR_002798. Please make sure this RRID suggestion is accurate before including in your manuscript.

Generated by SciScore.
SciScore only screens the text of the methods section; we apologize if the tool has missed items reported elsewhere in the paper or supplements.

Transparency

?	Open code	If you wrote code to analyze your data, please consider sharing the code in a public repository. This makes it easier for others to reproduce your analyses, and may also aid others seeking to analyze similar datasets.
?	Open data	If permitted, sharing data on a public repository with a persistent identifier (a DOI or accession number) can improve reproducibility and make it easier for other scientists to expand on your work. Papers with open data are cited more often than papers without open data. Some institutions have an expert who can provide advice on data sharing.
✓	Self-acknowledged limitations	"From a methodological perspective, this highlights an important limitation of standard experimental designs: because most studies rely on singly housed animals, current estimates of FAA may overstate the behavioral impact of food entrainment under more naturalistic social conditions. The use of implanted telemetry devices enables the study of these rhythms in group-housed animals while preserving individual-level resolution, providing a more ecologically relevant measurement for investigating food-entrained behavior. Several limitations should be considered. While our study focused on locomotor activity and body temperature, additional measures of metabolic rate, hormonal signaling, and neural activity within food-entrainable circuits will be required to define underlying mechanisms. In addition, factors such as group composition, social hierarchy, and cage density may further influence FAA and warrant systematic investigation. All experiments were conducted under standard vivarium tem..."
?	Conflict of interest statement	It is important to supply a conflict of interest statement, even if there are none to declare, to transparently present the research.
?	Funding statement	It is important to supply a funding statement because it makes the funding source and potential conflicts of interests transparent.
n/a	Registration statement	A registered protocol makes the planned research project more transparent and can

	protect against bias. Please consider registering future confirmatory studies.

Generated by ODDPub, limitation-recognizer, rtransparent, trial-identifier.

Figures

✓	No bar graphs of continuous data	ℹ Thank you for not using bar graphs to present continuous data. Many different datasets and data distributions can lead to the same bar graph, and the actual data may suggest different conclusions from the summary statistics alone.
?	Bar graphs with dot plots	ℹ Thank you for showing individual data points on your bar graphs. We recommend removing the bars and showing only a dot plot. The resource linked in the information symbol illustrates several reasons why dot plots are better than bar graphs with dot plots.
✓	No rainbow color maps	ℹ Thank you for not using rainbow colormaps. Rainbow color maps distort readers' perception of the data by introducing visual artifacts and are not accessible to colorblind readers.

Generated by JetFighter, Barzooka.
