# Supplementary material for: Social context suppresses food anticipatory activity and associated thermoregulation in mice
Source: PLoS One. 2026 Aug 28;21(8):e0356499. doi: 10.1371/journal.pone.0356499 (PMC13524294; doi:10.1371/journal.pone.0356499)
Supplement: Peer review history

# Peer review: Decision Letter 0

**DOI:** 10.1371/journal.pone.0356499.r001

**Authors:** Nicholas S. Foulkes

14 Jun 2026

PONE-D-26-20478

Social Context Suppresses Food Anticipatory Activity and Associated Thermoregulation in Mice

PLOS One

Dear Dr. Steele,

Thank you for submitting your manuscript to PLOS ONE. After careful consideration, we feel that it has merit but does not fully meet PLOS ONE’s publication criteria as it currently stands. Therefore, we invite you to submit a revised version of the manuscript that addresses the points raised during the review process.

This manuscript has now been reviewed by 4 experts in the field as well as the ScreenIT system. They all gave a generally positive evaluation: The use of an individualised telemetry system was seen as a important innovation for exploring the origin of Food Anticipatory Activity within a social context and all recognised the interest in and value of these findings however, they have also raised a series of concerns that need to be addressed before this work can be considered suitable for publication.

A few points as examples:

The complexity of the "social environment" in this work seems to have been "glossed over" to some extent. Group housing may have multiple, complex effects on animals including affecting stress levels as well as impacting on hierarchies. These points need to be considered and discussed in much more detail.

The observed hypothermia bouts were seen as a potentially important finding but these were not discussed or examined in detail. Furthermore, the merits of considering links between FAA and body temperature changes need to be discussed in more detail.

Care should be taken when extrapolating from the results. In some has verged on pure speculation.

The presentation of Figures and some data can certainly be optimised.

An ethics statement is needed in the Methods section.

This list is NOT exhaustive and the authors need to carefully consider all the comments of the Reviewers.

Please submit your revised manuscript by Jul 31 2026 11:59PM. If you will need more time than this to complete your revisions, please reply to this message or contact the journal office at plosone@plos.org. When you're ready to submit your revision, log on to https://www.editorialmanager.com/pone/ and select the 'Submissions Needing Revision' folder to locate your manuscript file.

Please include the following items when submitting your revised manuscript:

- A letter that responds to each point raised by the academic editor and reviewer(s). You should upload this letter as a separate file labeled 'Response to Reviewers'.
- A marked-up copy of your manuscript that highlights changes made to the original version. You should upload this as a separate file labeled 'Revised Manuscript with Track Changes'.
- An unmarked version of your revised paper without tracked changes. You should upload this as a separate file labeled 'Manuscript'.

If you would like to make changes to your financial disclosure, please include your updated statement in your cover letter. Guidelines for resubmitting your figure files are available below the reviewer comments at the end of this letter.

If applicable, we recommend that you deposit your laboratory protocols in protocols.io to enhance the reproducibility of your results. Protocols.io assigns your protocol its own identifier (DOI) so that it can be cited independently in the future. For instructions see: https://journals.plos.org/plosone/s/submission-guidelines#loc-laboratory-protocols. Additionally, PLOS ONE offers an option for publishing peer-reviewed Lab Protocol articles, which describe protocols hosted on protocols.io. Read more information on sharing protocols at https://plos.org/protocols?utm_medium=editorial-email&utm_source=authorletters&utm_campaign=protocols.

As the corresponding author, your ORCID iD is verified in the submission system and will appear in the published article. PLOS supports the use of ORCID, and we encourage all coauthors to register for an ORCID iD and use it as well. Please encourage your coauthors to verify their ORCID iD within the submission system before final acceptance, as unverified ORCID iDs will not appear in the published article. Only the individual author can complete the verification step; PLOS staff cannot verify ORCID iDs on behalf of authors.

We look forward to receiving your revised manuscript.

Kind regards,

Nicholas S. Foulkes, D.Phil

Academic Editor

PLOS One

Journal Requirements:

When submitting your revision, we need you to address these additional requirements.

1. Please ensure that your manuscript meets PLOS ONE's style requirements, including those for file naming. The PLOS ONE style templates can be found at

https://journals.plos.org/plosone/s/file?id=wjVg/PLOSOne_formatting_sample_main_body.pdf and

https://journals.plos.org/plosone/s/file?id=ba62/PLOSOne_formatting_sample_title_authors_affiliations.pdf

2. To comply with PLOS One submissions requirements, in your Methods section, please provide additional information regarding the experiments involving animals and ensure you have included details on (1) methods of sacrifice, (2) methods of anesthesia and/or analgesia, and (3) efforts to alleviate suffering.

3.  We note that the grant information you provided in the ‘Funding Information’ and ‘Financial Disclosure’ sections do not match.

When you resubmit, please ensure that you provide the correct grant numbers for the awards you received for your study in the ‘Funding Information’ section

4. Thank you for stating the following financial disclosure:

“Funding was provided by the National Institute of General Medical Sciences of the National Institutes of the National Institutes of Health under award number R16GM145576 and by the National Institute of Diabetes and Digestive and Kidney Diseases of the National Institutes of Health DK132736 to ADS”

Please state what role the funders took in the study.  If the funders had no role, please state: "The funders had no role in study design, data collection and analysis, decision to publish, or preparation of the manuscript."

If this statement is not correct you must amend it as needed.

Please include this amended Role of Funder statement in your cover letter; we will change the online submission form on your behalf.

5. Please include your full ethics statement in the ‘Methods’ section of your manuscript file. In your statement, please include the full name of the IRB or ethics committee who approved or waived your study, as well as whether or not you obtained informed written or verbal consent. If consent was waived for your study, please include this information in your statement as well.

6. Please include captions for your Supporting Information files at the end of your manuscript, and update any in-text citations to match accordingly. Please see our Supporting Information guidelines for more information: http://journals.plos.org/plosone/s/supporting-information.

If the reviewer comments include a recommendation to cite specific previously published works, please review and evaluate these publications to determine whether they are relevant and should be cited. There is no requirement to cite these works unless the editor has indicated otherwise.

[Note: HTML markup is below. Please do not edit.]

Reviewers' comments:

Reviewer's Responses to Questions

**Comments to the Author**

1. Is the manuscript technically sound, and do the data support the conclusions?

Reviewer #1: Yes

Reviewer #2: Yes

Reviewer #3: Yes

Reviewer #4: Yes

Reviewer #5: Partly

**********

2. Has the statistical analysis been performed appropriately and rigorously?

Reviewer #1: Yes

Reviewer #2: Yes

Reviewer #3: Yes

Reviewer #4: Yes

Reviewer #5: No

**********

3. Have the authors made all data underlying the findings in their manuscript fully available?

The PLOS Data policy

Reviewer #1: Yes

Reviewer #2: Yes

Reviewer #3: Yes

Reviewer #4: Yes

Reviewer #5: No

**********

4. Is the manuscript presented in an intelligible fashion and written in standard English?

Reviewer #1: Yes

Reviewer #2: Yes

Reviewer #3: Yes

Reviewer #4: Yes

Reviewer #5: Yes

**********

Reviewer #1: This manuscript presents an interesting and technically sound descriptive study demonstrating that group housing suppresses food anticipatory activity (FAA) and associated preprandial temperature rhythms in mice. The use of implanted telemetry devices enabling individual-level measurements in socially housed mice represents a notable methodological strength. However, several conceptual and presentation issues should be addressed before publication.

Major Comments

1. Observation versus mechanism. The data convincingly demonstrate that group housing attenuates FAA and associated thermal rhythms. However, several mechanistic interpretations currently extend beyond what is directly supported by the presented data. In particular, the discussion of thermoregulatory demand, energetic state, and integrated physiological mechanisms should be framed more cautiously as hypotheses or possible interpretations rather than demonstrated mechanisms. The present study is strongest as a descriptive physiological report, and the conclusions should remain closely aligned with the experimental evidence.

2. Interpretation of “social context.” Group housing changes multiple environmental and physiological variables simultaneously, including social interaction, cage density, thermal microenvironment, stress levels, and potential dominance hierarchy effects. Therefore, the conclusions should be phrased carefully as effects of group housing/social housing conditions, rather than attributing the findings specifically to social interaction or thermoregulatory mechanisms alone.

3. Body temperature as an FAA-related parameter. The body temperature data are valuable and support the behavioral findings; however, body temperature should not be treated as equivalent to FAA itself. The manuscript would benefit from clearer distinction between locomotor FAA and associated physiological outputs such as preprandial thermal rhythms. In addition, because the study measures subcutaneous temperature rather than core body temperature, the limitations and potential influence of thermal microenvironment should be more clearly acknowledged.

4. Figure organization and temporal alignment. The current arrangement of Figure panels (A–N) makes comparison between locomotor activity and temperature somewhat difficult. A vertically aligned two-column layout (e.g., activity in one column and temperature in the other) may improve readability and better emphasize the parallel suppression of both outputs. In addition, the x-axis of panels C–J is currently centered around the nocturnal active phase. Since the principal biological phenomenon under investigation is FAA during ZT4–6, it would be more effective to center the representative traces around the preprandial anticipatory window.

Minor Comments

1. “ZT Time” is redundant. Because ZT already stands for “Zeitgeber Time,” the figure labels should be revised to either “ZT” or “Zeitgeber Time.”

2. Statistical reporting. The manuscript is generally well analyzed statistically. However, consistent formatting of statistical notation and spacing throughout the manuscript would improve readability.

3. Language and presentation. The manuscript is generally well written in standard scientific English. Only minor editorial revisions are required.

Reviewer #2: This is an automated report for PONE-D-26-20478. This report was solicited by the PLOS One editorial team and provided by ScreenIT.

ScreenIT is an independent group of scientists developing automated tools that analyze academic papers. A set of automated tools screened your submitted manuscript and provided the report below. Each tool was created by your academic colleagues with the goal of helping authors. The tools look for factors that are important for transparency, rigor and reproducibility, and we hope that the report might help you to improve reporting in your manuscript. Within the report you will find links to more information about the items that the tools check. These links include helpful papers, websites, or videos that explain why the item is important. While our screening tools aim to improve and maintain quality standards they may, on occasion, miss nuances specific to your study type or flag something incorrectly. Each tool has limitations that are described on the ScreenIT website. The tools screen the main file for the paper; they are not able to screen supplements stored in separate files. Please note that the Academic Editor had access to these comments while making a decision on your manuscript. The Academic Editor may ask that issues flagged in this report be addressed. If you would like to provide feedback on the ScreenIT tool, please email the team at ScreenIt@bih-charite.de. If you have questions or concerns about the review process, please contact the PLOS One office at plosone@plos.org.

Reviewer #3: This manuscript addresses a genuine and underappreciated gap in circadian biology — the near-total absence of FAA studies conducted under socially relevant housing conditions. Using subcutaneously implanted wireless telemetry ("nanotags"), the authors tracked individual locomotor activity and body temperature in both singly and group-housed C57BL/6J mice during time-restricted feeding. The core finding — that social housing suppresses FAA and blunts preprandial temperature rises — is biologically meaningful and methodologically enabled by a clever use of individualized telemetry in a group setting. The paper is generally well-written and the logic is sound. However, several methodological and analytical issues warrant attention before acceptance.

Comment 1: Group composition is partially confounded

Each group-housed cage contained two implanted and two non-implanted mice. The non-implanted animals serve no described experimental purpose yet share the social and thermal environment. Their presence may influence the behavior of implanted animals (e.g., huddling partners, dominance dynamics), and the reader has no information about whether these "filler" mice received any monitoring. This design choice should be explicitly justified, and the authors should address whether the non-implanted mice were screened for health or weight changes throughout the study.

Comment 2: Social hierarchy and individual variation are unaddressed

Group housing in male mice, particularly C57BL/6J, routinely produces dominance hierarchies that influence stress hormones, body weight, and activity levels. Given that 6/12 single-housed males showed hypothermic bouts — a striking degree of individual variation — it is unknown whether dominant vs. subordinate animals differ in FAA expression within group-housed cages. No within-cage individual comparisons are reported. This warrants at minimum an expanded discussion of how social hierarchy could confound or modulate the reported suppression of FAA in grouped animals.

Comment 3: Delta temperature metric requires clearer justification

The authors define delta temperature as the maximum value within the ZT6 hour minus the minimum within the ZT24 hour, derived from 5-minute bins. This operationalization is sensitive to outliers within those one-hour windows, as a single anomalous bin could disproportionately influence the daily delta value. A brief justification for this approach over alternatives — such as hourly means or a broader averaging window — would improve methodological transparency and reproducibility.

Comment 4: Figure 2C shows a significant difference on Day 0 before feeding restriction begins

The Results text describes Day 0 activity and temperature profiles as "broadly similar" between single and group-housed males, yet Figure 2D displays asterisks on the temperature trace, indicating statistically significant differences at baseline before any feeding manipulation was introduced. This discrepancy between the text and the figure is not adequately addressed in the Results section. Pre-existing baseline differences between housing groups could complicate the interpretation of subsequent changes as being driven by the time-restricted feeding protocol rather than by housing condition alone, and should be explicitly discussed.

Comment 5: Figure 1N legend contains an error

The legend for Figure 1N describes the delta temperature metric as "ZT24–7," while the Methods section defines it as the maximum temperature at ZT6 minus the minimum at ZT24, and Figure 2N correctly labels it "ZT24–6." The endpoint in the Figure 1N legend should be corrected to ZT6 for consistency with the Methods and other figures.

Comment 6: The hypothermia finding is striking but underexplored

The observation that 6 out of 12 single-housed males exhibited acute hypothermic bouts (subcutaneous temperature below 28°C) during the mid-light phase is notable and reported with appropriate statistics via Fisher's exact test. However, this finding receives only brief mention and is relegated to a supplemental figure. Key questions remain unaddressed: Do these animals show correspondingly stronger FAA? Does hypothermia precede or follow anticipatory activity bouts, suggesting an energetic cost of FAA in isolated animals? This finding has the potential to meaningfully support the paper's central argument that FAA reflects an energetically demanding physiological state, and it deserves integration into the main Discussion rather than remaining in supplemental material alone.

Reviewer #4: Manuscript ID: PONE-D-26-20478

This is a very interesting manuscript reporting food-anticipatory locomotor activity and subcutaneous body temperature in social-housed conditions in mice. The authors measured individual locomotor activity and body temperature changes in response to timed-food restrictions in single-housed and group-housed mice using wireless implants. The anticipatory locomotor activity was suppressed in group-housed mice.

I believe the study of the effect of social housing on anticipatory behavior is interesting and warrants publication in the journal. However, I have a few concerns.

My main concern is that food intake appears to have increased during the RF period compared to Day 0. However, it is well established that RF induces calorie restriction (reduced food intake) in mice. It is not clear how the food intake is measured?

Is this the daily intake on days 0, 7, 14, and 20? Or is it the daily average of the 7-day food intake? How was it measured for individual mouse in the grouped-housed condition?

Minor comments:

1. Include the ethics statement in the material and methods section

2. Adding the feeding window in the figures, either as a bar below the graph or as a distinct shaded area, would make it easier for the readers to interpret the data.

Reviewer #5: This is an interesting and potentially publishable study. It addresses an important gap by testing whether social housing alters food anticipatory activity and related thermoregulation in mice, using implanted nanotags to monitor individuals in group-housed cages. The main finding, that group housing suppresses FAA especially in males, is clear and relevant.

However, several issues require clarification before the conclusions can be fully supported.

1. Lines 368–375. The main concern is whether individual mice or cages should be treated as the experimental unit. Although nanotags provide individual-level measurements, mice within the same group-housed cage share food access, social environment, microclimate, hierarchy, odor cues, and handling. The authors state that group-housed cages contained four mice and that individual animals were used as the unit of analysis, but cage may be the appropriate unit for the housing manipulation. The authors should report the number of cages per group and either reanalyze key outcomes using cage as a random effect or cage-level means, or justify animal-level independence.

2. Lines 376–380. Food intake is used to argue that caloric intake does not drive the phenotype, but in group-housed cages intake appears to have been measured per cage rather than per individual. Authors should clarify how intake was measured and normalized, and whether implanted and non-implanted animals had comparable food access.

3. Lines 143–150. Group-housed males weighed more than singly housed males despite similar reported food intake. This finding should be discussed more explicitly, as it may support the energetic-demand model but also raises questions about thermogenic costs, hypothermia, anticipatory activity, or unequal food access.

4. Lines 160–164. The hypothermic bouts in singly housed males are potentially important. The authors should define these events more rigorously, report their duration and frequency, and test whether animals showing hypothermia disproportionately drive the FAA phenotype. A sensitivity analysis excluding these animals would be useful.

5. Lines 198–228. The sex-specific interpretation should be softened. The abstract states that social housing suppresses FAA in both sexes, but the female effect appears weaker and transient, whereas the male effect is more robust and sustained. The abstract and discussion should reflect this distinction.

6. Lines 401-410. The statistical section needs more detail. The authors should specify which variables were analyzed with ANOVA versus mixed-effects models, how repeated measures and missing data were handled, and whether assumptions were checked. A summary table of analyses would improve reproducibility.

**********

what does this mean?). If published, this will include your full peer review and any attached files.

If you choose “no”, your identity will remain anonymous but your review may still be made public.

**Do you want your identity to be public for this peer review?**  For information about this choice, including consent withdrawal, please see our Privacy Policy

Reviewer #1: No

Reviewer #2: No

Reviewer #3: No

Reviewer #4: No

Reviewer #5: No

**********

[NOTE: If reviewer comments were submitted as an attachment file, they will be attached to this email and accessible via the submission site. Please log into your account, locate the manuscript record, and check for the action link "View Attachments". If this link does not appear, there are no attachment files.]

To ensure your figures meet our technical requirements, please review our figure guidelines: https://journals.plos.org/plosone/s/figures

You may also use PLOS’s free figure tool, NAAS, to help you prepare publication quality figures: https://journals.plos.org/plosone/s/figures#loc-tools-for-figure-preparation.

NAAS will assess whether your figures meet our technical requirements by comparing each figure against our figure specifications.

Attachment Submitted filename: report_10.64898+2026.04.10.717725.pdf

# Author comment: Author response to Decision Letter 1

**DOI:** 10.1371/journal.pone.0356499.r002

3 Jul 2026

Please see attached point-by-point response document.

Attachment Submitted filename: Response to reviewer comments.docx

# Editor report: Decision Letter 1

**DOI:** 10.1371/journal.pone.0356499.r003

**Authors:** Nicholas S. Foulkes

4 Aug 2026

Social Context Suppresses Food Anticipatory Activity and Associated Thermoregulation in Mice

PONE-D-26-20478R1

Dear Dr. Steele,

We’re pleased to inform you that your manuscript has been judged scientifically suitable for publication and will be formally accepted for publication once it meets all outstanding technical requirements.

Within one week, you’ll receive an e-mail detailing the required amendments. When these have been addressed, you’ll receive a formal acceptance letter and your manuscript will be scheduled for publication.

An invoice will be generated when your article is formally accepted. Please note, if your institution has a publishing partnership with PLOS and your article meets the relevant criteria, all or part of your publication costs will be covered. Please make sure your user information is up-to-date by logging into Editorial Manager at Editorial Manager® and clicking the ‘Update My Information' link at the top of the page. For questions related to billing, please contact billing support.

If your institution or institutions have a press office, please notify them about your upcoming paper to help maximize its impact. If they’ll be preparing press materials, please inform our press team as soon as possible -- no later than 48 hours after receiving the formal acceptance. Your manuscript will remain under strict press embargo until 2 pm Eastern Time on the date of publication. For more information, please contact onepress@plos.org.

Kind regards,

Nicholas S. Foulkes, D.Phil

Academic Editor

PLOS One

Additional Editor Comments (optional):

The authors have now substantially revised and improved thier manuscript. All of the primary concerns raised during the first review process have now been addressed satisfactorily.

Reviewers' comments:

# Editor report: Acceptance letter

**DOI:** 10.1371/journal.pone.0356499.r004

**Authors:** Nicholas S. Foulkes

PONE-D-26-20478R1

PLOS One

Dear Dr. Steele,

I'm pleased to inform you that your manuscript has been deemed suitable for publication in PLOS One. Congratulations! Your manuscript is now being handed over to our production team.

At this stage, our production department will prepare your paper for publication. This includes ensuring the following:

* All references, tables, and figures are properly cited

* All relevant supporting information is included in the manuscript submission,

* There are no issues that prevent the paper from being properly typeset

You will receive further instructions from the production team, including instructions on how to review your proof when it is ready. Please keep in mind that we are working through a large volume of accepted articles, so please give us a few days to review your paper and let you know the next and final steps.

Lastly, if your institution or institutions have a press office, please let them know about your upcoming paper now to help maximize its impact. If they'll be preparing press materials, please inform our press team within the next 48 hours. Your manuscript will remain under strict press embargo until 2 pm Eastern Time on the date of publication. For more information, please contact onepress@plos.org.

You will receive an invoice from PLOS for your publication fee after your manuscript has reached the completed accept phase. If you receive an email requesting payment before acceptance or for any other service, this may be a phishing scheme. Learn how to identify phishing emails and protect your accounts at https://explore.plos.org/phishing.

If we can help with anything else, please email us at customercare@plos.org.

Thank you for submitting your work to PLOS One and supporting open access.

Kind regards,

PLOS ONE Editorial Office Staff

on behalf of

Dr. Nicholas S. Foulkes

Academic Editor

PLOS One
